# The systems biology format converter

**DOI:** 10.1186/s12859-016-1000-2

**Published:** 2016-04-05

**Authors:** Nicolas Rodriguez, Jean-Baptiste Pettit, Piero Dalle Pezze, Lu Li, Arnaud Henry, Martijn P. van Iersel, Gael Jalowicki, Martina Kutmon, Kedar N. Natarajan, David Tolnay, Melanie I. Stefan, Chris T. Evelo, Nicolas Le Novère

**Affiliations:** EMBL European Bioinformatics Institute, Wellcome Trust Genome Campus, CB10 1SD, Cambridge, Hinxton UK; The Babraham Institute, Babraham Campus, CB22 3AT, Cambridge, UK; Department of Bioinformatics, BiGCaT, Maastricht University, Maastricht, 6229 The Netherlands; California Institute of Technology, Division of Biology and Biological Engineering, Pasadena, 91125 CA USA

**Keywords:** Converter, Format, Systems biology, SBML

## Abstract

**Background:**

Interoperability between formats is a recurring problem in systems biology research. Many tools have been developed to convert computational models from one format to another. However, they have been developed independently, resulting in redundancy of efforts and lack of synergy.

**Results:**

Here we present the System Biology Format Converter (SBFC), which provide a generic framework to potentially convert any format into another. The framework currently includes several converters translating between the following formats: SBML, BioPAX, SBGN-ML, Matlab, Octave, XPP, GPML, Dot, MDL and APM. This software is written in Java and can be used as a standalone executable or web service.

**Conclusions:**

The SBFC framework is an evolving software project. Existing converters can be used and improved, and new converters can be easily added, making SBFC useful to both modellers and developers. The source code and documentation of the framework are freely available from the project web site.

**Electronic supplementary material:**

The online version of this article (doi:10.1186/s12859-016-1000-2) contains supplementary material, which is available to authorized users.

## Background

Computational representations of pathways and models lie at the core of systems biology research [1]. Formats have been designed to encode these complex knowledge representations, either as community standards or as formats specific to a software tool [2]. Different formats are preferentially used to address specific problems or use different approaches, thus limiting interoperability. However, one often needs to use several tools and approaches to answer a biological question, or to reuse existing pathways and models in different contexts. Many format converters have been written over the years. Often, several converters between the same formats are developed independently by different groups. This results in a duplication of efforts and waste of time, energy and money. The different converters may be inconsistent, leading to different results. In addition, being developed by one person or one team, those software tools tend to go unmaintained while the formats they are covering keep evolving. Finally, some of these converters are embedded in larger pieces of software, which hinders their use.

To overcome these challenges, the Systems Biology Format Converter (SBFC) software provides an open source modular and extensible framework to potentially support conversion between any two formats using a single executable or web service.

## Implementation

**Fig. 1:**
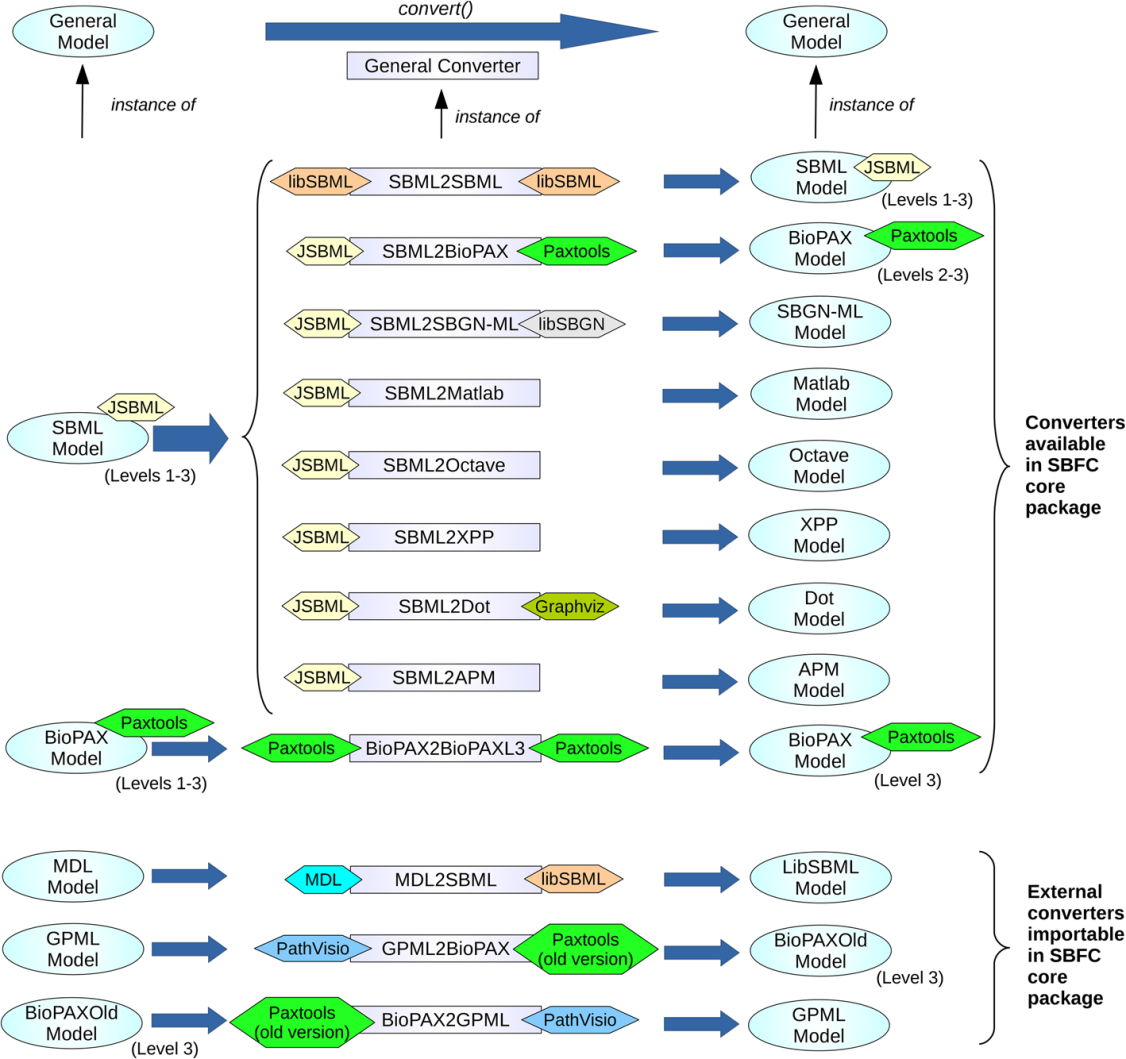
SBFC overview. Overview for the software package SBFC. At the SBFC core a general converter translates a general model into another. Instantiations of general model and general converter are easily implemented in SBFC, providing users with a wide range of options for converting between specific model formats. Software libraries for importing or exporting model formats can be reused by different converters. For instance, the converter SBML2BioPAX currently uses the software libraries JSBML to import an SBML model, and PAXTOOLS to export it

SBFC was built to support rapid implementation and integration of new converters. Therefore, it was designed with a high degree of modularity. At the core of the software are the **GeneralModel** interface and the **GeneralConverter** abstract class. The former is used for data exchange and describes the operations that every input or output format object must implement to be processed by SBFC. The latter represents the generic algorithm for converting one format into another. An overview of the SBFC framework is provided on Fig. 1.

To add a new format, a developer must simply implement the **GeneralModel** interface, which provides some methods to read and write the format to file or string. Adding a new converter requires extending the **GeneralConverter** class and implementing the *GeneralModel convert(GeneralModel model)* method, where the *model* parameter is the input format that needs to be converted and the returned *GeneralModel* object is the new converted format. For instance a converter A2B translating from a file formatted as *model A* to a file formatted as *model B*, requires the definition of two classes **ModelA** and **ModelB** implementing the **GeneralModel** interface. The class converter **A2B** must extend the abstract class **GeneralConverter** and implement the method *GeneralModel convert(GeneralModel model)*. This method will receive an input object named *model*, whose dynamic type is **ModelA**. The object returned by this method will have dynamic type **ModelB**.

**Fig. 2:**
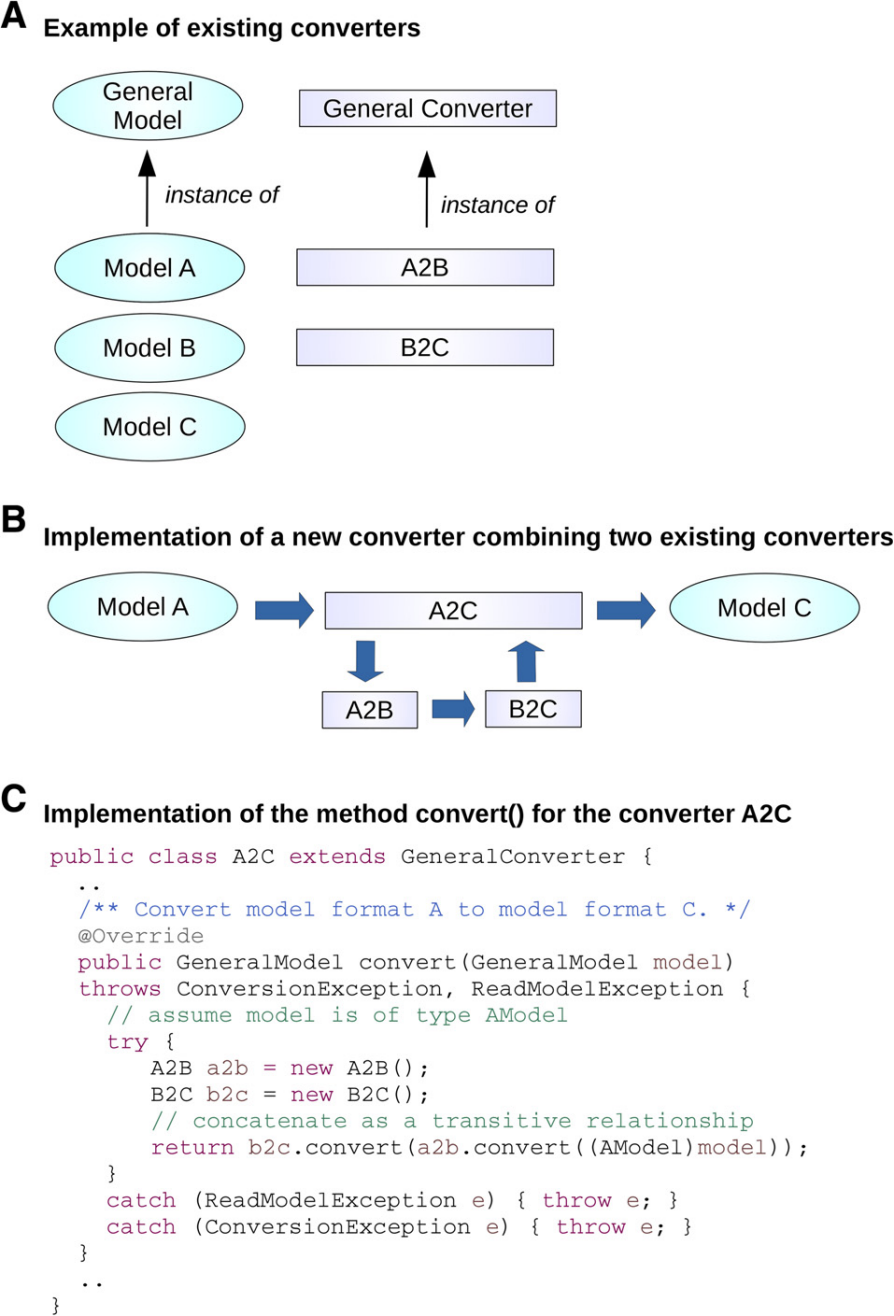
Creation of complex converters. **a** In this scenario, three existing formats (A, B, and C) and two converters (A2B and B2C) are considered. Each of the A, B and C classes represents a different format and implements the interface GeneralModel. The converters extend the GeneralConverter class and translate from A to B, and from B to C respectively. **b** A new converter A2C translating from A to C, can be added effortlessly by invoking the method convert() implemented in the converters A2B and B2C. **c** Java source code illustrating the implementation of the method convert() for the converter class A2C

Because all SBFC format classes are implementations of the **GeneralModel** interface, it is possible to create new converters re-using existing converters by simply invoking the generic *convert()* method for each existing converter (Fig. 2). The *convert()* method in the new converter A2C is implemented by calling the *convert()* methods in the converters A2B and B2C, respectively (source code for all classes is available in the SBFC manual).

SBFC is developed using the Java programming language. However, if an existing converter is developed in a programming language other than Java, it is still possible to create a new SBFC converter that will invoke the existing converter using the Java *Runtime exec()* method. This approach can be used for invoking any external program or command without having to re-write the full converter. Once the converter is integrated into the framework, it can be used and combined effortlessly with other converters (source code available in the SBFC manual). A potential disadvantage of this approach is the loss of interoperability when using operating system-dependent code. The advantage is that the specific SBFC converters directly rely on the development of the original external converters reducing code duplication.

Each format is identified by an identifiers.org URI [3] or an internet media type. If none of them exists, the developers of the format and converter classes must agree on an identifier (URI) for this format. SBFC allows multiple classes implementing the GeneralModel interface for a given format, using different tools to read and write models. All classes should return the same value for the *getURI()* method. For instance, the implementation of a converter for the Systems Biology Markup Language [4] may rely on JSBML [5], libSBML [6], or a DOM document structure [7]. This design can be advantageous when 1) a given library does not read a version of a format properly; 2) a converter was written with an old or newer version of a library that has a different API; or 3) high performance is required (e.g. improving the implementation for file processing). At the beginning of a conversion, the converter checks that the value returned by the *getURI()* method of the input **GeneralModel** is a URI of a format it does support. If the converter recognises the format URI, the generic write methods (*modelToString()* or *modelToFile(String fileName)*) are used in order to retrieve the file content.

## Results

### Available formats and converters

The SBFC project already implemented support for several formats and developed several converters. The following format classes are provided:

- *APMModel* for the APMonitor Modelling Language (APM). APMonitor is an optimization software for mixed-integer and differential algebraic equations [8];
- *BioPAXModel* for BioPax, format to exchange descriptions of biomolecular pathways, including reaction and interaction networks [9];
- *DotModel* for the Dot format, that encodes graph descriptions used by the open source graph visualisation software GraphViz [10] to generate multiple image formats (e.g. PNG, JPEG, etc);
- *GPMLModel* for the format used by the pathway drawing and analysis tool PathVisio [11] and the pathway database WikiPathways [12];
- *MDLModel* for the format used by the single particle simulator MCell [13];
- *OctaveModel* for Octave and MatLab m-file formats, encoding mathematical models usable by the modeling environments GNU Octave [14] and MatLab;
- *SBGNMLModel* for SBGN-ML format, a format to encode graphical maps in the Systems Biology Graphical Notation [15];
- *SBMLModel* for SBML [4], a format encoding mathematical models;
- *XPPModel* for XPP format, encoding mathematical models usable by the numerical analysis software XPPAUT [16].

This core set of model formats was based on the set of converters internally used by the BioModels project [17]. As SBML is central for this resource, the focus was on implementing converters from SBML to other formats. The following converters listed by class name are currently available: SBML2SBML, SBML2APM, SBML2BioPAX, SBML2SBGNML, SBML2Matlab, SBML2Octave, SBML2XPP, SBML2Dot. SBFC also supports conversions between SBML model annotations urn:miriam [18] and Identifiers.org URL [3] with the converters URN2URL and URL2URN. The Systems Biology community has been developing additional converters, including MDL2SBML, GPML2BioPAX, BioPAX2BioPAXL3 (converting from BioPAX Level 1 and 2 to BioPAX Level 3).

### Running SBFC as a standalone application

SBFC can be used as a standalone application and executed via a basic graphical user interface or the command line. The general command to convert one model into another on the command line is provided by the **Converter** class within the package org.sbfc.converter. The syntax for this generic command is:

- *java org.sbfc.converter.Converter [InputModelClass] [ConverterClass] [ModelFile]*

For instance, a model in SBML format can be converted to Octave format using the command:

- *java org.sbfc.converter.Converter SBMLModel SBML2Octave model.xml*

Bash and bat scripts are provided for most converters. This removes the need to specify the Java classpath, the input class, and converter classes. The previous conversion becomes:

- .*/sbml2octave.sh my-sbml-model.xml*

Users can specify a folder name as parameter, instead of a model file. In this case, the script will try to convert all xml files found in the folder.

**Fig. 3:**
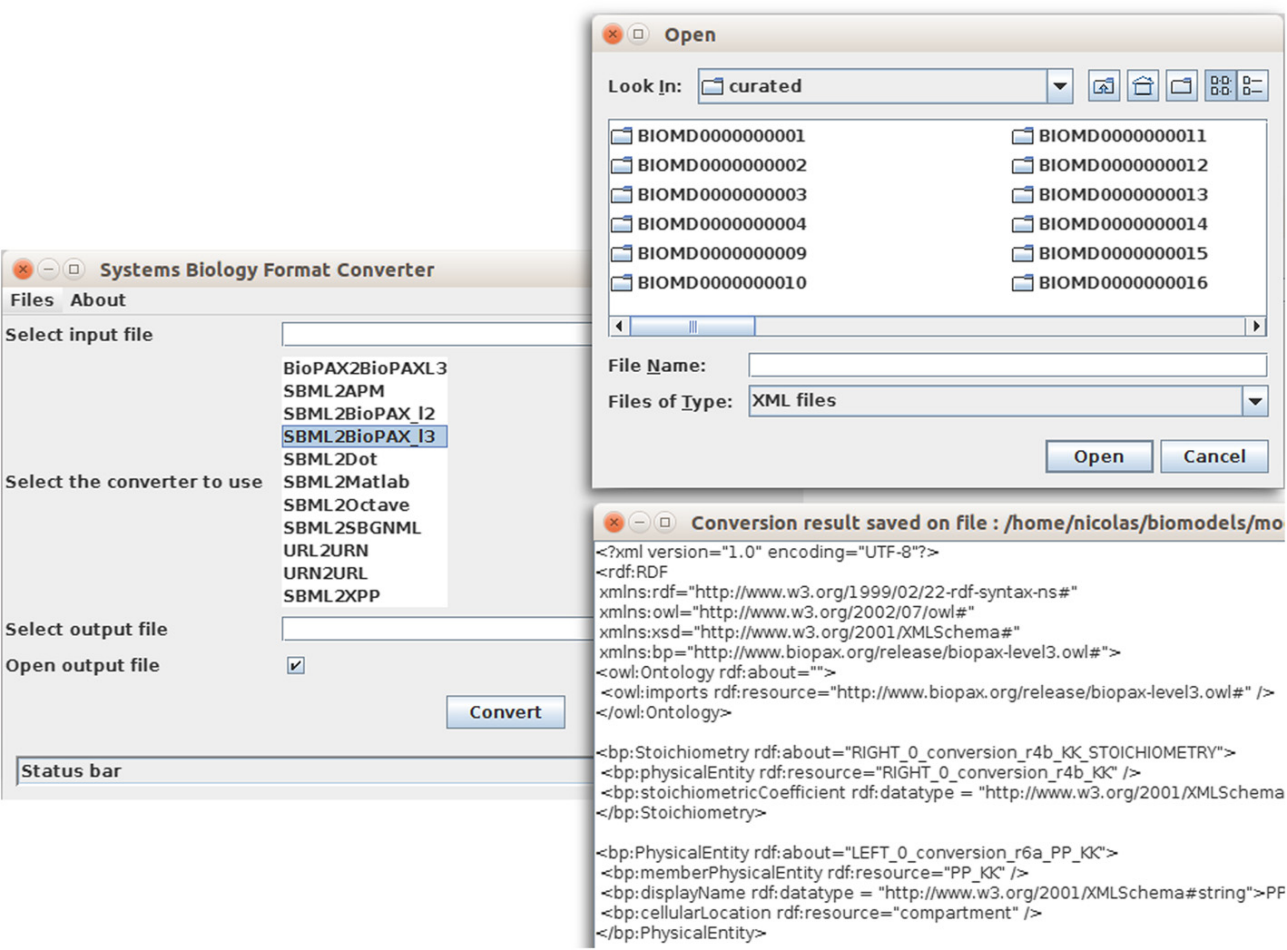
SBFC standalone graphical user interface. A simple GUI is provided to quickly convert a file. The user can browse and select a file, choose a converter, select a destination for the resulting conversion an launch the job. The result can optionally be displayed in a separate window

If the number of files to be converted is limited, a simple standalone GUI can also be used (Fig. 3). The general syntax is:

- *java -jar sbfc-x.y.z-standalone.jar*

On most systems, a double click on the Jar is sufficient but some scripts are also provided to help users to launch the GUI:

- .*/sbfConverterGUI.sh* for Linux or *sbfConverterGUI.bat* on Windows

This GUI can be embedded in any 3^rd^ party Java software with the command:

- *ConverterGUI.getConverterGuiInstance().setVisible(true);*

### Running SBFC online

**Fig. 4:**
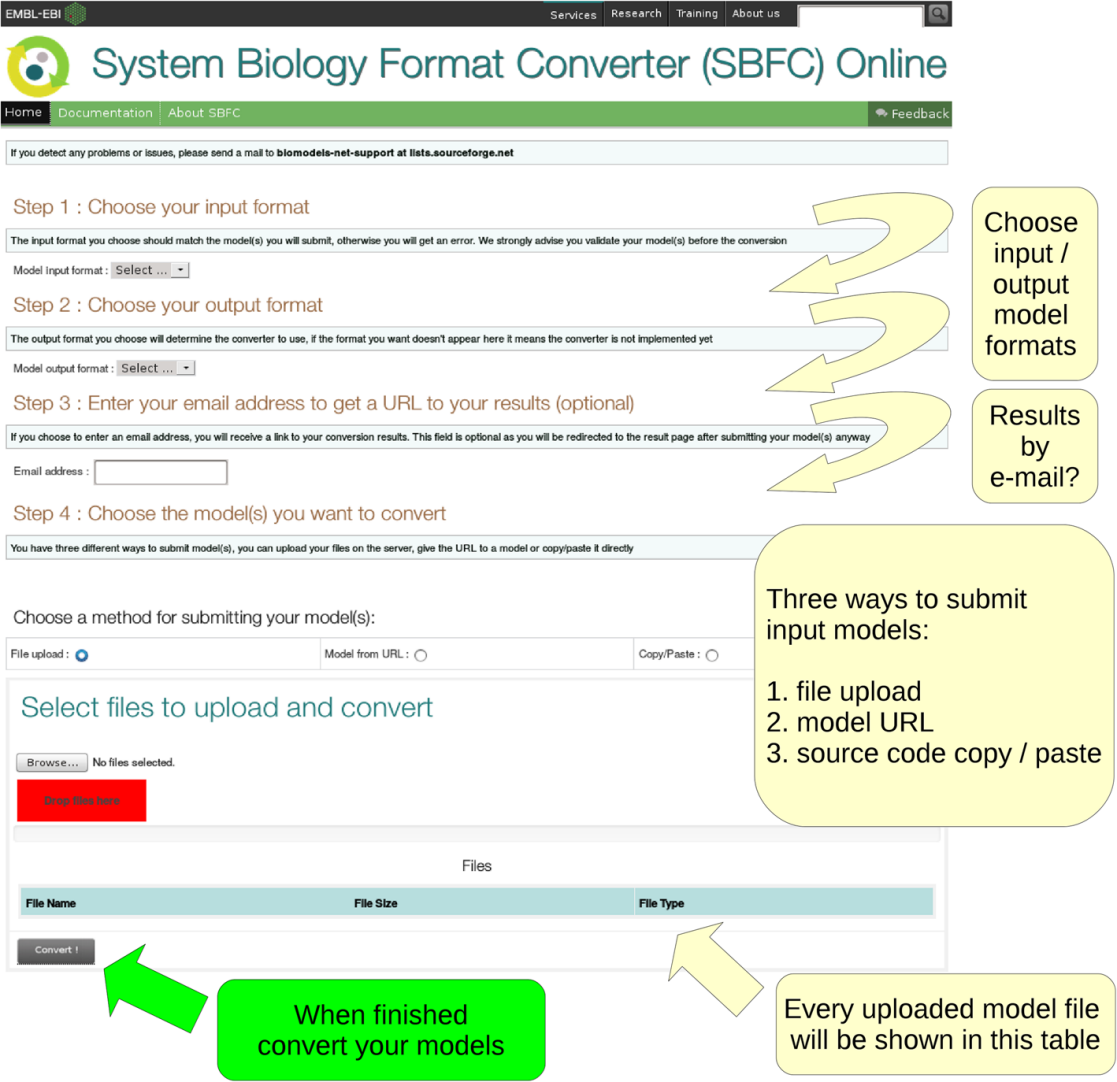
SBFC online. After selecting the input format, the available output format list is updated accordingly. A short description about the selected model format appears once an item from the combobox is selected. Three methods to submit the files to be converted are offered: *file upload*, *model URL*, and *copy/paste*. Files can be uploaded via a dialog window or by simply dragging and dropping them on the red box. Several files can be loaded before launching the conversion. The result page presents a list of all conversion jobs. Until the job is completed, a red “pending” box is displayed. The box turns to a green “complete” when the resulting files are available

To provide easy access to SBFC, a web application has been deployed at the European Bioinformatics Institute (EMBL-EBI) [19]. The web page is used in four successive steps as shown in Fig. 4. The user must first specify the input and output model formats. Once the input format is selected, the list of possible output formats is updated depending of the converters available on the system. An e-mail address can be optionally provided to receive a URL link to these results. Finally, the documents to convert can be selected. This can be done via three methods: file upload, model URL or copy/paste. Once a conversion is launched, the user is redirected to a result page. All the jobs launched during a browser session will be displayed. The results can be downloaded for 72 h. It is worth noting that for privacy purposes neither the original nor the converted models are kept on the servers for a period longer than 72 h.

The SBFC Online application can be downloaded from the Sourceforge website and installed locally as described in the SBFC developer manual.

### Running SBFC through Web services

For automatic and programmatic access, SBFC provides REST like webservices using the HTTP POST method. For java users, an helper class *SBFCWebServiceClient* provides an API that simplifies the use of the Web Services. Depending on user requirements, two types of methods can be selected for submitting conversion jobs. The first type performs blocking calls. The methods *submitAndGetResultFromFile*, *submitAndGetResultFromURL* and *submitAndGetResultFromString* start a conversion job and wait until the model is converted and returned from the web server. For large models, the conversion process can last several minutes, particularly if the cluster load is high. The second type of methods use asynchronous, nonblocking calls. The methods *submitJobFromFile*, *submitJobFromURL* and *submitJobFromString* start a conversion job and immediately return a *ConversionId* object that contain some metadata about the job. The status of the job can be checked later with the *getJobStatus* method. When the returned status is ‘done’, the user can use the method *getConvertionResult* to retrieve the output file. An example of java code that launches several conversions through the SBFC Web Services is provided in the additional file *UsageExample.java* [see Additional file 1].

## Discussion

Novel formats are created on a regular basis in systems biology, due to the development of new software tools for building and analysing biological pathways, networks and models. While these developments are necessary for the progress of the domain, interoperability is a crucial challenge and conversion tools play a central role. In order to maintain feature rich and up to date code, as well as to limit redundant efforts, community efforts are need. SBFC is an attempt in that direction.

SBFC was implemented in Java in order to be fully portable on all operating systems provided with a Java virtual machine. This facilitates software adoption by users as these do not have to install platform-specific versions, and by developers who can easily integrate SBFC in their existing Java software if needed. The provided web services also allow software not implemented in Java to use SBFC easily.

SBFC architecture is sufficiently generic to permit rapid implementations and additions of new formats and converters. With this solid but flexible design, SBFC aims to make existing efforts to develop systems biology converters converge into a single community activity.

A roadmap including new features and converters to be developed in the near future is available [20]. Contribution from the community is needed to integrate converters more quickly. An important feature which is still in progress is the complete adoption of the OSGi framework [21] within SBFC. This implementation would reduce problems related to library conflicts between different converters. The future SBFC OSGi plugins would also enable a complete integration of SBFC as Cytoscape 3 [22] plugin.

## Conclusions

SBFC is a novel open source software that provides a generic Java-based architecture for converting between Systems Biology model formats (but not limited to those). It helps computational biologists to process or visualise their models using different software tools, and software developers to implement format conversion. We hope that new converters will be contributed in the future.

## Availability and requirements

Project name: Systems Biology Format Converter (SBFC) Project home page: http://sbfc.sourceforge.net/ Operating system(s): Platform independent Programming language: Java SE 6 or higher Other requirements: None License: GNU LGPL v2

## Additional file

Additional file 1 SBFC Web Services usage example. The file *UsageExample.java* presents some code using several methods of the *SBFCWebServiceClient* class to perform some conversions, retrieve the results and save them to local files. (JAVA 5 kb)

## Abbreviations

APM: APMonitor Modelling Language
BioPAX: Biological Pathway Exchange
GPML: Pathvisio Graphical Pathway Markup Language
MDL: MCell Modelling Description Language
SBFC: Systems Biology Format Converter
SBGN: Systems Biology Graphical Notation
SBML: Systems Biology Markup Language
URI: Uniform Resource Identifier
